# Use of a Nasal Cannula as a Preoxygenation Adjunct: A Randomized Crossover Study

**DOI:** 10.1155/2024/7873142

**Published:** 2024-09-05

**Authors:** Murphy Joel, Suvajit Podder, Savan Kumar Nagesh, Ramyatha Aithal, Aditya R. Devalla, Shaji Mathew

**Affiliations:** Department of Anaesthesiology Kasturba Medical College of Manipal Manipal Academy of Higher Education, Manipal 576104, Karnataka, India

## Abstract

**Background:**

Preoxygenation prior to induction of general anesthesia is intended to increase the oxygen reserve in the lungs. This technique delays the onset of hypoxemia during the placement of the tracheal tube.

**Objective:**

To observe the benefits of oxygen through nasal cannula when used as an adjunct during preoxygenation.

**Methods:**

We enrolled 30 healthy volunteers and conducted a sequence of six preoxygenation tests. These included 3-minute tidal volume breathing and 8 vital capacity breaths, with and without oxygen flowing through the nasal cannula as an adjunct. Subjects were kept at a supine position with a face mask on their faces. Their baseline vitals were measured and end-tidal O_2_ (ETO_2_) was recorded at the end of each test. The comfort of each technique was also assessed.

**Results:**

When comparing the efficacy of the two preoxygenation methods, we found that the addition of oxygen through the nasal cannula improved the efficacy of preoxygenation with both the 3-minute tidal volume breathing method and the 8 vital capacity method (*p* < 0.001). The three-minute tidal volume breathing technique had higher end-tidal oxygen when compared to the eight vital capacity breaths.

**Conclusions:**

The administration of oxygen through a nasal cannula during preoxygenation improves the efficacy of preoxygenation in healthy volunteers. Tidal volume breathing for three minutes achieves a higher end-tidal oxygen concentration compared to eight vital capacity breaths over one minute.

## 1. Introduction

Preoxygenation before induction and intubation is an accepted technique to delay the onset of hypoxemia during airway management. It helps to prolong the apnea time during tracheal intubation in patients undergoing general anesthesia with neuromuscular paralysis. The effectiveness of preoxygenation is primarily based on two measurable factors, namely, efficacy and efficiency. The efficacy of preoxygenation is measured based on the increase in the fraction of alveolar O_2_ (FAO_2_), the reduction in alveolar nitrogen (FAN_2_), and the increase in alveolar tension (PaO_2_), while efficiency is measured by the decrease in saturation (SaO_2_) during apnea [1]. Effective preoxygenation can be confirmed by an increase in end-tidal O_2_ (ETO_2_) more than 90% or an end-tidal nitrogen concentration of 5% [2]. Techniques such as head-up position, use of positive airway pressure, and alternate breathing circuits have been tried to improve the effectiveness of preoxygenation [3–6]. Apneic oxygen via nasal cannula (NC) has been advised as a part of difficult airway management to prolong apnea time and improve preoxygenation efficiency [7]. High flow oxygen through a NC has also been used for preoxygenation in the ICU and emergency departments. The use of NC as an adjunct to mask preoxygenation can introduce leaks during preoxygenation with a facemask, possibly reducing the efficacy of preoxygenation [8, 9]. We hypothesized that the addition of oxygen via nasal cannula as an adjunct to the standard preoxygenation technique would improve its efficacy. The primary objective of our study was to measure the ETO_2_ at the end of preoxygenation using various flows through the nasal cannula.

## 2. Methodology

This prospective randomized crossover trail was conducted in 30 healthy volunteers, aged 20–50 years, in a tertiary care center. Pregnant women and people with facial trauma or dysmorphism, BMI > 30 kg/m^2^, and anticipated difficult airways were excluded from the trail. Male volunteers were asked to have a clean-shaven beard for the study. Written informed consent was obtained from the volunteers who were willing to participate in the trail. The study was approved by the institutional ethics committee and registered with CTRI (no. CTRI/2020/04/024873).

Participants were randomized to undergo preoxygenation using two methods (tidal volume breaths for 3 minutes (3 min TVB) and 8 Vital capacity breaths (8 VCB) over 1 minute), with supplemental oxygen via NC at 3 flow rates (0 L/min; 5 L/min, and 10 L/min).

Thus, each participant underwent six sequences of preoxygenation.

1. 3 min TVB with NC 0 L/min
2. 3 min TVB with NC 5 L/min
3. 3 min TVB with NC 10 L/min
4. 8 VCB with NC 0 L/min
5. 8 VCB with NC 5 L/min
6. 8 VCB with NC 10 L/min

All participants completed all 6 methods of preoxygenation in a random order, based on a Latin square method. The sequence of preoxygenation was randomized by the author SP using an online balanced Latin square generator (https://damienmasson.com/tools/latin_square) to generate a 6 × 6 table. The order in which these sequences would be performed was then randomized for 30 participants using an online randomizer.

Subjects were placed in a supine position with a pillow under the occiput. Baseline heart rate, blood pressure, and ETO_2_ were documented. A closed anesthesia circuit (GE Carestation 650 with a 1.6-meter Limbo Circuit and 2 L bag) was flushed with 100% oxygen. The nasal cannula was attached to the auxiliary oxygen port of the anesthetic machine and placed over the nostrils of the participants. Preoxygenation (10 L/min) with anesthesia circuit and NC oxygen was started according to the sequence selected. Participants were asked to breathe normally for 3 minutes of tidal volume breathing and take slow deep breaths every 7 to 8 seconds for the 8 vital capacity breaths. The face mask was held by the investigators and adjusted to minimize leakage. This was confirmed by an adequate continuous waveform capnogram. The distortion of the waveform capnogram indicated an inadequate seal, and the mask seal would be adjusted to optimize the waveform as much as possible. Flows through NC were stopped before the last three breaths for 3 min TVB and the last breath for VCB to prevent the dilution of ETO_2_. The ETO_2_ value at the end of the preoxygenation period was documented (E-sCAiO gas analyzer with a D-Fend Pro water trap, GE Healthcare). Each method of preoxygenation was followed by a washout period, with subject breathing room air, until the baseline ETO_2_ value (±2%) was reached, as shown in supplementary material 1.

The comfort of the participant was assessed after each preoxygenation sequence using the Likert scale [10].

1. Very uncomfortable
2. Uncomfortable
3. Bearable
4. Comfortable
5. Very comfortable

Based on the reference study [11], a clinically significant difference of 5% and pooled standard deviation was taken as 7% to give an effect size of 0.35. A change in ETO_2_ of 5% was considered clinically significant cause it could increase the safe apnea time in an 80 kg male by 30 seconds. With a power of 0.8 and significance of 0.05, the calculated sample size for our study was 26. Thirty participants were enrolled to balance participants for five sets of six sequences.

Statistical analysis was performed using the Statistical Package for Social Sciences (SPSS) for Windows version 22.0 released in 2013. Armonk, NY: IBM Corp. Significance tests were performed with a univariate type III repeated-measures ANOVA with ETO_2_ as the dependent variable and preoxygenation trials as the within subjects variable. A Bonferroni correction to the *p* values was used for the post hoc test. The paired Student's *t*-test was used to compare the two preoxygenation techniques.

## 3. Results

**Figure 1:**
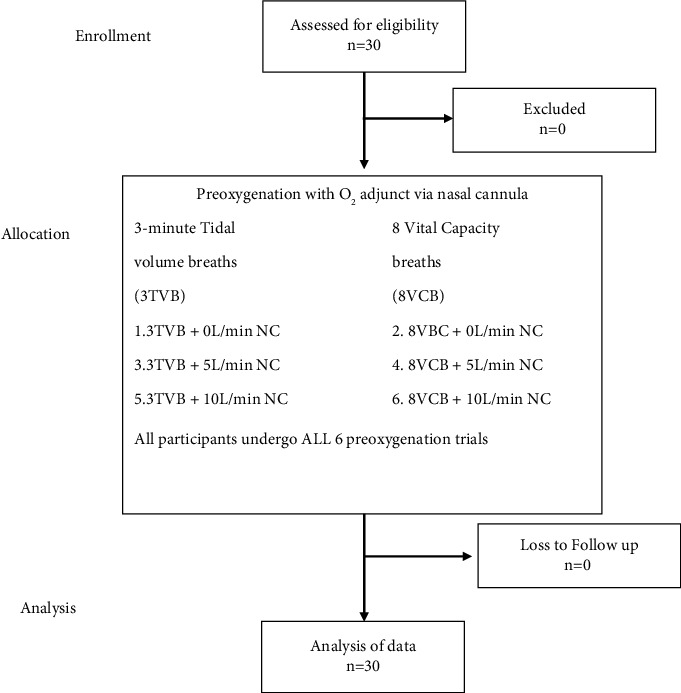
CONSORT flow diagram.

**Figure 2:**
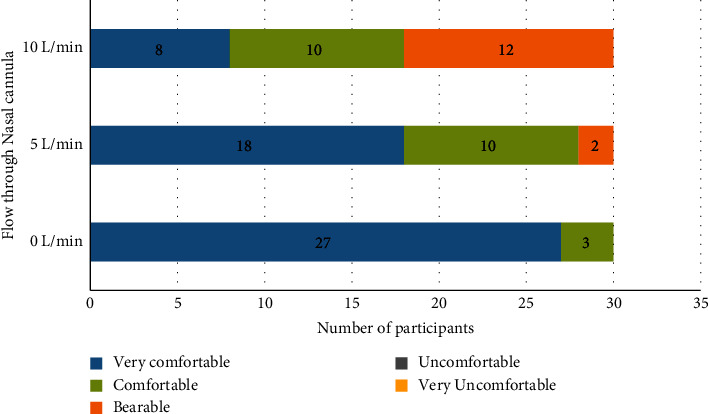
Participant comfort.

Thirty healthy volunteers were enrolled in this study. The CONSORT diagram describes the enrollment process (Figure 1). The mean age was 27 years and the mean BMI was 23.7 ± 2.5 kg/m^2^ (Table 1). When preoxygenated with 3 minute of TV breathing, the mean ETO_2_ increased from 81.90 ± 2.41% to 91.07 ± 1.7% as the flow increased from 0 L/min to 10 L/min, while preoxygenation with 8 vital capacity breaths over one minute technique, the mean ETO_2_ increased from 77.27 ± 2.26% to 87.87 ± 2.29% as the flow increased to 10 L/min (Tables 2 and 3). When comparing the efficacy of the two preoxygenation methods, we found that 3-minute TVB was better than the 8 VC method (Table 4, *p* < 0.001). Even with the use of a nasal cannula as an adjunct and 10 L/min of O_2_ flow, the mean ETO_2_ was higher with 3 min TV breathing (91.07% vs. 87.87%), which was clinically and statistically significant (*p* < 0.001). The comfort level of the participants with various flows of O_2_ through the nasal cannula was also documented. Flows of 10 L/min were uncomfortable or just bearable by most of the participants. Most subjects were comfortable with a 5 L/min flow through the nasal cannula (Figure 2).

## 4. Discussion

In our study, we compared the feasibility, effectiveness, and comfort of using nasal cannula as an adjunct to standard preoxygenation techniques in volunteers. We found that using nasal cannula as an adjunct improved the end-tidal oxygen concentration, irrespective of the preoxygenation technique used (3-minute tidal volume respiration and 8 vital capacity breaths over 1 minute).

Effective preoxygenation improves the safety of patients during airway manipulation by increasing the safe apneic time [12]. This is especially evident in certain subset of patients such as pediatric, pregnant women, obesity, and full stomach and patients with a difficult airway. The prevalence of unanticipated difficult airway is 0.9–1.9% [13]. The pediatric population is particularly susceptible to hypoxia during general anesthesia and sedation due to greater oxygen consumption and diminished functional residual capacity. It is reported that over 50% of critical events during the perioperative period in children are respiratory related [14]. Various techniques have been studied to improve the effectiveness of preoxygenation; breathing at different respiratory capacities, breathing circuits, and different oxygen flow rates [1, 8–15]. The use of nasal cannula as an adjunct to preoxygenation has previously been studied on volunteers. Bradley et al. concluded that the addition of supplemental oxygen through a nasal cannula did not improve ETO_2_ compared to preoxygenation with a bad mask valve device without leak [9]. McQuade et al. [11] in their study found addition of nasal cannula with oxygen flow rate at 5 L/min delays optimal preoxygenation. Both studies attributed these findings to a considerable leak introduced by the nasal cannula, which breaches the seal provided by the bag mask valve device. In our study, we ensured leak was minimal by adjusting the mask seal to minimize distortion in the continuous capnography waveforms. We were able to achieve similar ETO_2_ values (82%) in our study to those achieved by the former authors (79% and 84%) with a tight-fitting bag mask valve device and no leak, possibly demonstrating that a good mask seal can be achieved even with a nasal cannula in place. A study by Russell et al. [8] that also employed a good mask seal concluded that the use of supplemental oxygen at 5 L/min through the nasal cannula improved the mean ETO_2_ at the end of the 3-minute TV breathing method of preoxygenation.

We also evaluated the efficacy of nasal cannula adjunct during the 8 vital capacity (8VC) breathing technique of preoxygenation. We found that 3-minute TV breathing was better than 8 VC breaths over one minute as a preoxygenation technique, and the use of nasal cannula as an adjunct improved the ETO2 in both 3-minute TV and 8VC technique. Russell et al. found the addition of nasal cannula less effective while using the 8VC technique. Other studies also concluded that maximum ETO_2_ was not reached at the end of one minute while taking 8 VC breaths [8].

A recent systematic review and network meta-analysis compared various techniques to identify the most effective technique [16]. In their findings, Carvalho et al. suggest use of high flow nasal oxygen (HFNO) along with head up position as the most effective preoxygenation technique prior to induction of general anesthesia to prolong safe apnea time. They also rank the use of pressure support with head up position as the number one technique which can lead to the fastest rise in ETO_2_ (data for rise in ETO_2_ were unavailable for HFNO studies). The better efficacy of preoxygenation while using a high flow nasal oxygen could be due to the increased total oxygen flow rates, which provide positive airway pressure that can cause alveolar recruitment and reduce ventilation perfusion mismatches. Other possible explanations could be increased CO_2_ washout from the dead space by the high flow of oxygen, improving the FiO_2_ delivered. It also presents a continuous source of oxygen during the apnea period (apneic oxygenation), improving safe apnea time.

The use of nasal cannula as adjunct to the standard preoxygenation technique is an ingenious way to achieve the benefits of using a high flow nasal oxygen device in low resource settings. The addition of oxygen via nasal cannula as an adjunct to standard mask preoxygenation increases the overall oxygen flow (10–20 l/min), while ensuring proper mask seal can create positive airway pressure. Thus, this combination can help achieve effective preoxygenation in scenarios where access to HFNO devices is limited. The requirements to perform the preoxygenation techniques as in our study would be the standard anesthetic circuit, a nasal cannula, and an auxiliary oxygen source.

The use of nasal cannula as an adjunct to preoxygenation has been associated with discomfort although tolerable. In our study, we found that discomfort with the nasal cannula increased with increasing flow rates. A flow of 5 L/min was found to provide a balance between tolerability and effective preoxygenation (Figure 2). The findings of our study lead us to advocate the use of nasal cannula as an adjunct during preoxygenation.

There has been an advent of newer ultra-short-acting anesthetic agents such as remizolam, which have proved to be particularly useful in several perioperative settings (e.g., cardiac pts undergoing cardiac or noncardiac surgery and the pediatric population) [17, 18]. Use of such agents could help tolerate very high flows and improve preoxygenation comfort, thereby safely achieving benefits of preoxygenation with minimal patient discomfort.

Preoxygenation with 5 L/min oxygen through the nasal cannula may be sufficient to overcome any possible leaks imposed by the presence of the nasal cannula when a reasonably tight fit of the mask can be ensured. The nasal cannula can be left in place throughout airway management, providing an extra margin of safety. Other clinical scenarios where the nasal cannula could be useful are for emergency caesarean section. With limited induction and intubation time, the nasal cannula with 10 L/min oxygen flow and 8 VC breath may be able to provide adequate preoxygenation. Here, the discomfort of the higher flow is of comparatively shorter duration and hence patients might be more compliant.

Our trial is not free of limitations. The trial was carried out on healthy volunteers, which limits its validity in a clinical setting. The effectiveness of preoxygenation was not determined (only the efficacy was evaluated using ETO_2_) and the use of ETO_2_ as the end point of successful preoxygenation is inferior to the use of PaO_2_ while assessing the efficacy of preoxygenation. ETO_2_ was used as it is a noninvasive and commonly used technique of documenting end point of preoxygenation. Further studies in the clinical scenario and in special population (pregnancy, difficult airway) would strengthen the evidence for the use of nasal cannula as an adjunct for preoxygenation using standard face mask.

In conclusion, the administration of oxygen through a nasal cannula during preoxygenation improves the efficacy of preoxygenation in healthy volunteers. Tidal volume breathing for three minutes achieves a higher end-tidal oxygen concentration compared to eight vital capacity breaths for one minute.

## Figures and Tables

**Table 1:** Characteristics of the participants.

Age (years)	*n* = 30	Percentage
25–27	20	66.7
28–30	9	30
>30	1	3.3
Gender (M/F)	8/22	26.7/73.3
BMI (kg/m^2^) mean ± SD	23.7 ± 2.5	

Source: authors.

**Table 2:** Mean end-tidal O_2_ after 3 minutes of TV breaths (*n* = 30).

Intervention	Mean ETO_2_ (%) [min-max]	±SD	Comparison groups	*p* value^#^
0 L/min nasal cannula oxygen	81.90 [78–86]	2.41	0 L/min vs 5 L/min	<0.001
5 L/min nasal cannula oxygen	87.30 [82–91]	2.51	0 L/min vs 10 L/min	<0.001
10 L/min nasal cannula oxygen	91.07 [88–95]	1.70	5 L/min vs 10 L/min	<0.001

^#^ *p* value derived by repeated measures of ANOVA followed by Bonferroni's post hoc analysis. Source: authors.

**Table 3:** Mean end-tidal O_2_ after 8 vital capacity (VC) breaths (*n* = 30).

Intervention	Mean ETO_2_ (%) [min-max]	±SD	Comparison groups	*p* value^#^
0 L/min nasal cannula oxygen	77.27 [72–81]	2.26	0 L/min vs 5 L/min	<0.001
5 L/min nasal cannula oxygen	83.13 [78–88]	2.18	0 L/min vs 10 L/min	<0.001
10 L/min nasal cannula oxygen	87.87 [83–92]	2.29	5 L/min vs 10 L/min	<0.001

^#^ *p* value derived by repeated measures of ANOVA followed by Bonferroni's post hoc analysis. Source: authors.

**Table 4:** Comparison between 3 min TV breaths and 8 VC breaths over 1 min (*n* = 30).

Intervention		Mean (ETO_2_%)	±SD	*p* value
0 L/min nasal cannula oxygen	3 TV	81.90	2.41	<0.001^∗^
	8 VC	77.27	2.26	

5 L/min nasal cannula oxygen	3 TV	87.30	2.51	<0.001^∗^
	8 VC	83.13	2.18	

10 L/min nasal cannula oxygen	3 TV	91.07	1.70	<0.001^∗^
	8 VC	87.87	2.29	

^∗^Student's *t*-test. Source: authors.

## Data Availability

Data from this clinical study can be accessed by requesting the same from the corresponding author through e-mail (savankum@gmail.com).

## Additional Points

*What do We Know About the Problem?* Nasal oxygen is advised during anticipated difficult airway situations to provide apneic oxygenation. High flow nasal oxygen has also been used for preoxygenation. The addition of nasal cannula with low flow oxygen during preoxygenation as an adjunct to standard bag mask valve can have a positive or negative effect based on reduced dead space and higher FiO_2_ or leak and entrainment, respectively. *What Does This Study Contribute?* In healthy volunteers, the addition of O_2_ through nasal cannula to standard face mask techniques improved the end-tidal O_2_ at the end of the duration of preoxygenation.

## Supplementary Materials

Supplementary Materials Supplementary material 1: a flowchart showing example of one of the participants sequences of preoxygenation. The entire chronological steps that the participants underwent with 6 preoxygenation techniques are described. Supplementary material 2: the statistical formula used to calculate the sample size and the calculations done is described.
