# Supplementary material for: Use of a Nasal Cannula as a Preoxygenation Adjunct: A Randomized Crossover Study
Source: Anesthesiol Res Pract. 2024 Sep 5;2024:7873142. doi: 10.1155/2024/7873142 (PMC11392575; doi:10.1155/2024/7873142)
Supplement: Supplementary Materials — Supplementary material 1: a flowchart showing example of one of the participants sequences of preoxygenation. The entire chronological steps that the participants underwent with 6 preoxygenation techniques are described. Supplementary material 2: the statistical formula used to calculate the sample size and the calculations done is described. [file 7873142.f1.zip › Flowchart explaining the sequence of preoxygenation in a volunteer.docx]

Flowchart explaining the sequence of preoxygenation in a volunteer.

Sequence 2: **Preoxygenation technique** – 3 minute tidal volume breathing + O_2_ via nasal cannula at 0L/min

Sequence 1: **Preoxygenation technique** – 3 minute tidal volume breathing + O_2_ via nasal cannula at 5L/min

Sequence 3: **Preoxygenation technique** – 8 vital capacity breaths over 1 minute with O_2_ via nasal cannula at 0L/min

Sequence 4: **Preoxygenation technique** – 3 minute tidal volume breathing + O_2_ via nasal cannula at 10L/min

Sequence 5: **Preoxygenation technique** – 8 vital capacity breaths over 1 minute + O_2_ via nasal cannula at 10L/min

Sequence 6: **Preoxygenation technique** – 8 vital capacity breaths over 1 minute + O_2_ via nasal cannula at 5L/min

End of study
