# Supplementary material for: Use of a Nasal Cannula as a Preoxygenation Adjunct: A Randomized Crossover Study
Source: Anesthesiol Res Pract. 2024 Sep 5;2024:7873142. doi: 10.1155/2024/7873142 (PMC11392575; doi:10.1155/2024/7873142)
Supplement: Supplementary file 3 [file 7873142.f1.zip › Statistical formula used for calculating sample size.odt]

Statistical formula used for calculating sample size

A math equations and formulas

Description automatically generated with medium confidence

Source: SPSS

Pooled standard deviation (SD) = 7

Z (alpha) table value = 1.96                                                                    

Z (beta) table value = 0.84

Clinically significant difference (d) = 5

Number of repetitions (m) = 6

Required sample size (each group): 12.8
